# Decoding methods for neural prostheses: where have we reached?

**DOI:** 10.3389/fnsys.2014.00129

**Published:** 2014-07-16

**Authors:** Zheng Li

**Affiliations:** ^1^State Key Laboratory of Cognitive Neuroscience and Learning and IDG/McGovern Institute for Brain Research, Beijing Normal UniversityBeijing, China; ^2^Center for Collaboration and Innovation in Brain and Learning Sciences, Beijing Normal UniversityBeijing, China

**Keywords:** brain-machine interface, brain computer interface, decoding, neural prosthetic, neural engineering, multichannel recordings, signal processing

## Abstract

This article reviews advances in decoding methods for brain-machine interfaces (BMIs). Recent work has focused on practical considerations for future clinical deployment of prosthetics. This review is organized by open questions in the field such as what variables to decode, how to design neural tuning models, which neurons to select, how to design models of desired actions, how to learn decoder parameters during prosthetic operation, and how to adapt to changes in neural signals and neural tuning. The concluding discussion highlights the need to design and test decoders within the context of their expected use and the need to answer the question of how much control accuracy is good enough for a prosthetic.

## Introduction

The field of brain-machine interfaces (BMIs) for control of motor prostheses is quickly growing (Baranauskas, 2014, for other reviews, see Tehovnik et al., 2013; Kao et al., 2014). Research in decoders, the algorithms which translate neural signals to movement commands, has largely switched focus from improving control accuracy to resolving practical considerations of future clinical deployments of prostheses. The goal of this mini-review is to briefly highlight recent (2013 to mid-2014) advances in decoding methodology for extracellular signals recorded from motor areas of the brain. The review sections are organized by main research themes, corresponding to important questions and practical considerations. At the end, the importance of developing and testing decoders in realistic contexts and the question of how much control accuracy is “good enough” for a prosthetic are discussed.

## Algorithms for decoding

**Figure 1:**
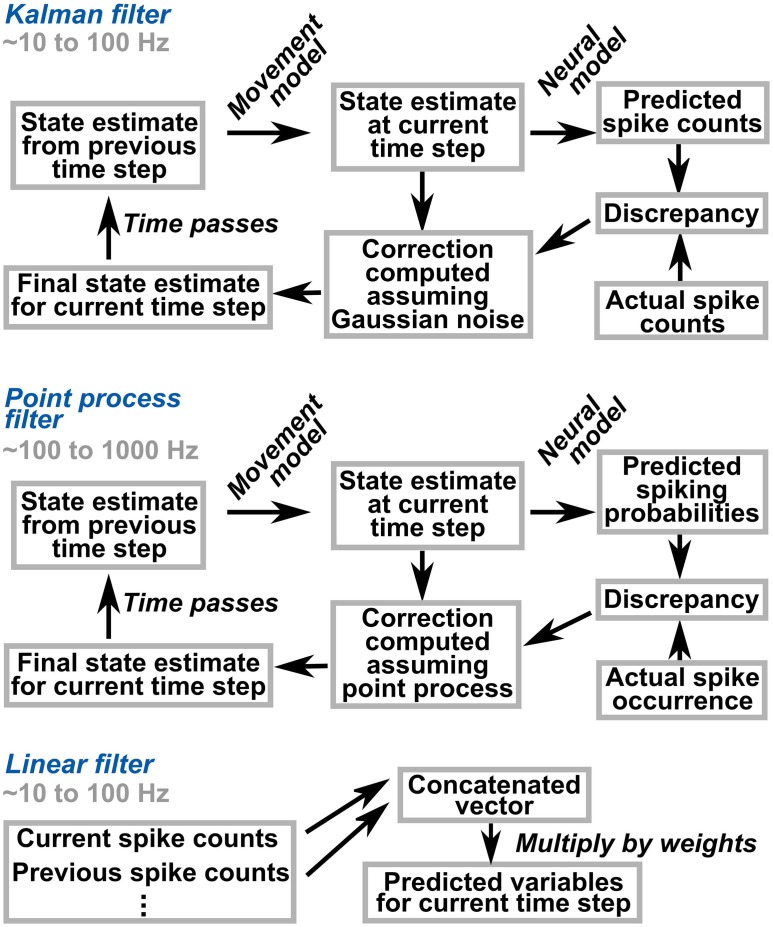
**Schematic illustration of popular decoding algorithms.** The Kalman and point process filters are based on the notion of a state, which holds the current estimates of the variables of interest. The state is related to neural activity through a neural model. Bayesian computations on the neural model, assuming a distribution for noise, permit probabilistic tracking of the state based on neural activity. In contrast, the linear filter is state-less; it linearly maps the recent history of neural activity to estimates of the variables of interest.

*Which algorithmic framework should we use for decoding?* Different algorithms offer different benefits. Figure 1 illustrates three commonly-used methods. The Kalman filter and point process filter are state-based (modeling temporal evolution) and probabilistic (modeling and estimating uncertainty). The linear filter, in contrast, is a linear transformation of neural data to the decoded variables, with the advantages of simplicity and execution speed.

The Kalman filter’s Gaussian noise model clearly does not match the data (spike counts), yet due to its accuracy and execution speed the method has remained popular since its first use by Wu et al. (2003) (Aggarwal et al., 2013; Chen et al., 2013; Dangi et al., 2013a,c; Homer et al., 2013; Ifft et al., 2013; Jarosiewicz et al., 2013; Kao et al., 2013; Merel et al., 2013; Wong et al., 2013; Zhang and Chase, 2013; Fan et al., 2014; Golub et al., 2014; Gowda et al., 2014; Homer et al., 2014). While point process filters (for a review, see Koyama et al., 2010) offer a more realistic noise model, their use in decoding is still relatively rare (Shanechi et al., 2013; Velliste et al., 2014; Xu et al., 2014), due in part to their heavier computational burden. Recently, Citi et al. (2013) extend point process methods to model refractory periods of neurons and allow for coarser time discretization by a factor of 10, which may ease this burden. However, one feature which current point process decoders lack is the ability to assign different amounts of noise or weights to different neurons.

Linear filtering, or discrete Wiener filtering, is fading in popularity. It is still used by some research groups, either because of its computational form (Badreldin et al., 2013) or when the research focuses on other aspects of decoding (Chen et al., 2013; Chhatbar and Francis, 2013; Philip et al., 2013; Suminski et al., 2013; Willett et al., 2013). A variant of the Wiener filter method, which passes the Wiener filter output through a fitted non-linear function to compute the final output, is also used (Flint et al., 2013; Scheid et al., 2013).

Several other methods have been used in the recent literature: kernel autoregressive moving average (KARMA; Wong et al., 2013), quantized kernel least mean square (Li et al., 2014), support vector machines (Cao et al., 2013; Xu et al., 2013; Wang et al., 2014), K-nearest neighbors (Brockmeier et al., 2013; Ifft et al., 2013; Xu et al., 2013), naïve Bayes (Bishop et al., 2014), and artificial neural networks (Chen et al., 2013; Mahmoudi et al., 2013; Pohlmeyer et al., 2014). All of these methods allow highly non-linear neural models.

## Variables to decode

*What values should a decoder predict?* Ideally, a prosthetic should offer accurate, intuitive control that works under all likely usage contexts. In most prior work, desired positions or velocities of end-effectors were decoded. Homer et al. (2013) proposed a method for combining decoded position and velocity, to avoid choosing between the two. The method defines a quantity Δ*r* as the difference between the decoded cursor position and the previous cursor position. The cursor position’s update is a linear combination of the decoded velocity and the decoded velocity vector rotated to the direction of Δ*r*.

Decoders aim to predict user intentions, and it is possible that intentions may be slightly different from the observed limb movements used for parameter fitting. Fan et al. (2014) showed that the heuristic for guessing user intention during online recalibration proposed by Gilja et al. (2012), and tested with human users in a study by Jarosiewicz et al. (2013), could also be applied to the initial training data for fitting decoder parameters. This method rotates the cursor’s velocity vector towards the target and zeroes the velocity when the cursor is in the target. Fitting Kalman filter parameters on these estimates of intended movements, instead of actual limb movements, could achieve comparable gains in accuracy as online recalibration using the same scheme (Fan et al., 2014).

Two recent studies have explored decoding torque values, to allow a prosthetic to interact with objects with mass more naturally. Chhatbar and Francis (2013) showed that hybrid neural control by both torque and position produced more natural movements in novel dynamic environments. Decoding of position and torque was performed via a Wiener filter applied to the largest 20 principal components of neural activity. The final angular accelerations of the prosthetic joints were calculated by a weighted sum of the accelerations implied by the predicted positions and torques. Suminski et al. (2013) decoded position and velocity as well as torques of a two-link virtual arm with a Wiener filter. The kinematic variables were converted to torques using a position-derivative controller and the results were combined linearly with the directly decoded torques to produce the final torque output.

Besides kinematics and forces, the target location of a reach may be directly decoded to improve the trajectory of the reach. Shanechi et al. (2013) presented a real-time, two-stage decoder which first decoded target location during an instructed delay period and then decoded reach trajectory during the reach period. The decoded target location served as the goal position for an optimal feedback controller that acted as the movement model of the point process trajectory decoder.

To address the need for BMI control of limbs with many degrees of freedom, an important consideration for clinical deployment, Wong et al. (2013) used principal component analysis to reduce the dimensions of limb movements. They showed that decoding principal-component-space kinematic variables with KARMA or Kalman filtering was more accurate than decoding canonical-space kinematic variables. Ifft et al. (2013) decoded the kinematics of both arms during a bimanual reaching task using an unscented Kalman filter which included variables for both arms in its state.

Besides the biomimetic approach of designing a prosthetic so that it can be controlled like a natural limb, another approach is to use operant conditioning of neuron ensembles (for a review, see Sakurai et al., 2014) to let the user learn to control a new, synthetic actuator. Though more initial training may be required, greater final control accuracy may be possible using this paradigm. Balasubramanian et al. (2013) used two groups of neurons from M1 to control reach and grasp, which were simplified to one dimension each. The neuron groups were chosen based on their stability and functional connectivity, and algorithmic assistance was given during the operant conditioning process to assist neuronal learning. Badreldin et al. (2013) developed an unsupervised method for non-biomimetic linear filter initialization. The method performs an eigendecomposition of the sample covariance matrix of the neural data. The eigenvectors provide a basis for the space of all possible linear filters that could be fitted from the data. They designed a cost function to choose a particular linear filter from this space, which probably differs from the filter which would have been fitted by supervised linear regression. Their cost function optimizes for characteristics such as low jitter and evenly distributed weights among neurons.

## Neural models

*How should we model neural activity?* Recent studies have explored the aspects of neural models beyond movement tuning, with the hope of improving user-friendliness. Considering that a system with high latency is difficult to control, Willett et al. (2013) trained a decoder to predict intended future movements to compensate for BMI system latency and shorten the feedback loop. To predict intended future movements, they fitted a linear filter using kinematic values which were temporally offset from the neural data.

In a clinical setting, it is important that a prosthetic can be turned off when not used, to avoid undesired movements. Aggarwal et al. (2013) classified behavioral states into baseline, reaction, movement, and hold using linear discriminant analysis (LDA) on local field potentials (LFPs), and then decoded arm, hand, and finger kinematics using a Kalman filter on spike signals. The position outputs were held constant when the behavioral state decoder predicted the baseline or hold state. Similarly, Velliste et al. (2014) used LDA to detect idle (resting) arm states and set velocity to zero during idle. The baseline or idle states in these studies could serve as the “off” mode for a prosthetic.

Xu et al. (2014) included ensemble firing history, in addition to the standard tuning to kinematic variables, in the neural model. This paradigm helps model the background activity that is unrelated to movements. They used parallel computation on graphics processing units to achieve real-time execution of a point process particle filter that used this model.

## Neuron selection

*Which neurons should we include in decoding?* BMI researchers have long sought ways to reduce computational load to decoders and noise in neural data by excluding irrelevant neurons. Several recent studies have provided tools for finding relevant neurons. Chen et al. (2013) used variational Bayesian inference to fit parameters for a linear filter, a state-space model, and a non-linear echo state network. Using priors which favor small parameters, the inference procedure generated sparse parameter fits, and the zeroes in the fitted parameters can be interpreted as the absence of tuning.

Cao et al. (2013) determined which neurons modulated for reach direction versus hand configuration during grasping by using mutual information. In another study from the same group, Xu et al. (2013) proposed a supervised metric learning algorithm to optimize decoding of hand grasp configuration. Their gradient descent algorithm maximizes the difference between inter-class and intra-class distances while regularizing by the L1 norm, resulting in sparse weights which indicate relevance.

Also using a supervised approach, Brockmeier et al. (2013) proposed a method for computing a linear dimensionality reduction which maximizes the information between the class labels and the projected neural data. The low dimensional data can be used for visualization or decoding via distance-based methods such as K-nearest neighbors. Brockmeier et al. (2013) proposed an improved method that only uses inner products between inputs, allowing non-linear dimensionality reduction via the kernel trick. Their kernel metric learning algorithm aims to make data points with the same class labels lay close together in the output space.

## Movement models

*How can we design movement models to assist decoding?* Kalman and point process filters include movement models which can encode prior beliefs about how variables change over time. Cleverly engineering these models may make prostheses easier to control. Two studies examined how to improve the user’s ability to stop a BMI cursor when desired. Golub et al. (2014) designed a speed-dampening Kalman filter which modifies the movement model to decrease speed when fast changes in movement direction are detected, with the goal of allowing a quick change in direction to signal the desire to stop (a “hockey stop”). Using a different approach, Velliste et al. (2014) added a separate speed variable, independent of the Cartesian velocity variables, to the state space of a point process filter. This speed term dynamically adjusts the filter’s movement model error covariance so that smaller changes in position and velocity are allowed when the decoded speed is smaller.

In a general examination of movement models, Gowda et al. (2014) analyzed the linear models typically used in past studies and found that some may harbor hidden attractor points, to the detriment of controllability. They also point out that specific coefficients in movement model matrices parameterize the speed-accuracy tradeoff.

## Learning

*How can we improve decoder parameters during decoding?* To handle poor initial parameter fits or changes in neural tuning after practice, continuous learning of decoder parameters may be required in a clinical device. There has been much recent work, mostly from Jose Carmena’s Lab (for a review, see Carmena, 2013), on improving decoder parameter fits during BMI operation, called closed-loop decoder adaptation. They adapted Kalman filter parameters via stochastic updates based on the likelihood gradient (Dangi et al., 2013a), provided tools for analysis of adaptive methods (Dangi et al., 2013b), and applied adaptation to decoding of LFPs (Dangi et al., 2013c).

Information about the target locations of reaches can help improve the parameter learning process. Kowalski et al. (2013) proposed an algorithm which uses the joint estimation paradigm (augmenting tuning parameters into the state space), combined with the “reach state equation” (Srinivasan et al., 2006) as a way to incorporate target location in decoder recalibration. Similarly, Shanechi and Carmena (2013) designed a dual filtering method which uses the target location to assist movements towards the target. The method provides the target location, assumed known, to a linear-quadratic-Gaussian optimal feedback controller which acts as the movement model of the point process decoder. A second point process filter updates the decoder parameters.

In Suminski et al.’s (2013) study, incongruence between decoded kinematics and torques were used as an error signal for recalibration. The differences between the decoded position (and velocity) and the virtual arm’s endpoint position (and velocity), as computed via the decoded torques, were used as an error signal to update torque decoder parameters via gradient descent.

Merel et al. (2013) modeled co-adaptation in BMIs as two agents (encoder and decoder) optimizing with respect to each other, under linear-quadratic-Gaussian assumptions. They derive a novel decoder update step which anticipates what the future encoder will be and updates with respect to that, instead of the current encoder. They show that this “one step ahead” update rule reduces error faster in simulations.

## Signal stability and adaptation

*Are neural signals stable over long time periods?* There has been controversy as to whether updating of decoder parameters is required for long-term prosthetic usability. Recent studies have analyzed stability of signals over long time spans. Flint et al. (2013) and Scheid et al. (2013) showed that multiunit spiking activity can be stable over more than six months and LFPs can be stable for almost a year. Wang et al. (2014) found signal instability and concluded that LFPs allowed more accurate offline reconstruction than single- and multi-unit signals 1–2 years post implantation. Perge et al. (2013) found significant intra-day changes in neural firing rates and concluded that 85% of these changes were likely due to physiological mechanisms.

*If decoder updates are needed, how can we improve the accuracy of updates?* Recent studies have proposed heuristics to improve adaptation. Zhang and Chase (2013) used two extensions to a dual-Kalman filter. First, they updated baseline firing rates of neurons using a moving window. Second, they normalized the velocity provided to the parameter updater so that the median absolute velocity matches that of the initial training data. Kao et al. (2013) proposed a firing rate normalization that also includes a regularization term that penalizes neurons with low firing rates. They also showed that dimensionality reduction via principal component analysis improves robustness to neuron loss.

Besides updating baseline firing rates via windowed estimates, other methods for tracking baseline changes have been proposed. Bishop et al. (2014) found that most changes occur between days. They designed a classifier for movement direction using the naïve Bayes algorithm and a hierarchical model; baseline firing rates are inferred each day while the class-specific parameters and the prior distributions for the baseline firing rates are learned once on initial training data. Homer et al. (2014) designed a probabilistic algorithm for detecting infrequent, rapid changes in baseline firing rates under the Kalman filtering framework. Their method first performs a forward stepwise search for neurons which have changed in baseline firing rate and then determines the magnitude of changes.

Using a reinforcement learning approach to adaptation, two studies from the same group (Mahmoudi et al., 2013; Pohlmeyer et al., 2014) showed that an actor-critic reinforcement learning BMI that uses Hebbian learning on an artificial neural network decoder’s weights could learn weights from scratch and maintain decoding accuracy despite shuffling, loss, or gain of neurons, using only a one-bit feedback signal. In another study from the same group, Prins et al. (2013) decoded a one-bit reward signal from nucleus accumbens by clustering spike counts with *k*-means.

## Discussion

As researchers focus more on practical hurdles to clinical deployment of neural prostheses, it becomes more and more important to develop and test BMI decoders in the contexts in which actual prostheses will be used, i.e., to control artificial limbs, natural limbs via functional electrical stimulation (Moritz et al., 2008; Ethier et al., 2012; Nishimura et al., 2013), or computer cursors in graphical user interfaces. By using more realistic contexts, questions such as which variables to decode or which algorithms are sufficiently fast can be answered empirically. Realistic contexts may also uncover new considerations and obstacles to overcome.

An important question which has been thus far neglected in the field is how much control accuracy is enough? Full restoration of human ability in terms of movement accuracy may come at computational and other costs, e.g., number of recording channels, which likely trade off against other figures of merit of a prosthetic system. While we should continually endeavor to improve BMI technology, from a practical standpoint, we should also answer the question of how much control is good enough, so that engineers can design systems with clear requirements in mind.

## Conflict of interest statement

The author declares that the research was conducted in the absence of any commercial or financial relationships that could be construed as a potential conflict of interest.
